# Optimizing Clinical and Cost Outcomes for Patients on Enteral Nutrition Support for Treatment of Exocrine Pancreatic Insufficiency: *Proceedings from an Expert Advisory Board Meeting*

**DOI:** 10.1089/pop.2019.0042

**Published:** 2019-05-10

**Authors:** Joseph I. Boullata, Janice L. Clarke, Archie Stone, Alexis Skoufalos, David B. Nash

**Affiliations:** ^1^ Department of Nutrition Sciences, Drexel University, Philadelphia, Pennsylvania.; ^2^ Clinical Nutrition Support Services, Hospital of the University of Pennsylvania, Philadelphia, Pennsylvania.; ^3^ Jefferson College of Population Health, Philadelphia, Pennsylvania.; ^4^ Alcresta Therapeutics, Inc., Newton, Massachusetts.

**Optimizing Clinical and Cost Outcomes for Patients on Enteral Nutrition Support for Treatment of Exocrine Pancreatic Insufficiency: *Proceedings from an Expert Advisory Board Meeting***

Joseph I. Boullata, PharmD, RPh, Janice L. Clarke, RN, Archie Stone, PhD, Alexis Skoufalos, EdD, and David B. Nash, MD, MBA

*Population Health Management* Supplement Policy *Population Health Management* publishes supplements that (*a*) discuss new technologies, theories, and/or practice, and (*b*) serve as enduring materials to disseminate information from conferences and special meetings. Supplements that discuss new technologies, theories, and/or practices are subject to peer review.

Acknowledgment The Expert Advisory Board Meeting and written report based on the proceedings were funded by an educational grant to the Jefferson College of Population Health from Alcresta Therapeutics, Inc. The content and opinions expressed herein are those of the authors and the Advisory Board presenters and participants.

## Editorial

### Treatments targeting small patient populations: an important conversation continues

David B. Nash, MD, MBA

Without doubt, lowering prices while increasing appropriate access to pharmaceuticals will remain a hot topic among health policy makers in the foreseeable future. In particular, the ongoing debate over the value of drugs and technologies designed to treat rare conditions will continue to draw close attention from both the public sector and mass media.

The Food and Drug Administration (FDA) defines a rare condition as one that affects fewer than 200,000 Americans.^1^ Generally, these conditions are considered to be severe in nature, progressive, degenerative, life-threatening, or chronically debilitating. Drugs developed to target rare conditions are sometimes referred to as orphan drugs. The Orphan Drug Act of 1983 incentivized pharmaceutical companies to develop new drugs for rare diseases and extended those incentives to existing drugs that could be approved and marketed for different indications and settings.^1^

As the development rate of orphan drugs and “niche” technologies continue to grow, the discussion tends to focus solely on the high price tag rather than meaningful efficacy and value for patients who are affected by these conditions and the potential cost savings associated with improved condition management. As a result, new products – even those associated with incrementally better clinical and quality of life outcomes than traditional approaches – are frequently viewed with skepticism.

This supplement offers insights into the complex issues involved in developing, showing effectiveness, demonstrating value, and ultimately delivering a life-changing new product to a small but vulnerable patient population. The new product described herein was developed specifically for patients suffering from exocrine pancreatic insufficiency (EPI); that is, diseases and conditions that affect the pancreas – hereditary conditions such as cystic fibrosis (CF) and acquired conditions such as chronic pancreatitis. Because of a lack of adequate pancreatic digestive enzymes, patients with EPI experience clinical symptoms related to malabsorption of fat. Many of these patients must rely on enteral nutrition (EN) to avoid malnutrition – especially patients with acute pancreatitis and/or pancreatic cancer, children with CF, patients in intensive care units (ICUs), patients with intestinal failure, and infants in neonatal ICUs.

None of the currently available pancreatic enzyme replacement therapies (PERT) are formulated for or indicated for use in patients receiving EN for several reasons. PERT capsules are not FDA approved for use with EN formulas. The “work-around” of adding crushed capsules to EN formula is contrary to practice guidelines and interrupts feedings by clogging the tube. Further, the capsules are not indicated for *overnight* EN because their activity peaks at 30 minutes and wanes thereafter.^2^ Most importantly, even when PERT capsules are administered in large doses, there is no evidence of normal fat absorption in patients receiving EN. (In fact, patients receiving EN were excluded from participating in the clinical trials that led to FDA approval of PERTs.) Consequently, many patients with EPI who follow this regimen continue to struggle nutritionally and experience clinical symptoms related to malabsorption of fats.

Developed to optimize treatment for the population of tube-fed patients with fat malabsorption, RELiZORB (immobilized lipase) is a novel hybrid technology – an in-line digestive cartridge designed specifically for hydrolyzing fats in enteral formulas. Easily administered without risk of clogging feeding tubes, the product has been shown to break down more than 90% of fats in EN formulas throughout overnight feedings.^3^ Ample real-world clinical evidence has shown improved tolerability and improved fatty acid absorption with positive outcomes reported in terms of weight gain and increased patient compliance with oral nutrition.^4^ In fact, Cystic Fibrosis Centers using this technology to treat CF patients receiving EN have documented actual weight gain and increased body mass index (BMI).^5–7^

We've all heard it said that technology generates value only if the health benefit outweighs the cost. Herein lies the next hurdle – getting a firm grasp on costs. The direct costs associated with traditional EN are significant ($80 to $200 per day for formula, tube feeding supplies, and PERT), and the outcomes achieved are suboptimal (CF patients who receive EN achieve only 60% of normal fat levels). Like patients with other EPI-related conditions, hospitalization becomes necessary when malabsorption becomes malnutrition. In-hospital costs of EN include medical professional time spent crushing and adding PERTs to feeding bags, unclogging feeding tubes, and addressing unresolved patient symptoms.

Like many innovative products, this one doesn't fit the typical payer reimbursement model. Although the FDA was clear in classifying the cartridge as a medical device, there has been ongoing debate around reimbursement (ie, whether to classify this as a medical device [because of the cartridge delivery mechanism] or a drug [because of the immobilized lipase delivered via the cartridge]). Payers consistently reimburse providers for standard therapies – even those that, like traditional EN regimens, often fail to achieve the intended goal. In this case, use of a new technology may optimize EN, enable patients to achieve nutritional goals, reduce or eliminate the need for PERT during tube feedings, and ultimately reduce avoidable costs associated with frequent hospitalizations and increased length of stay.

As I see it, targeted innovative therapy is the new reality, one with unimaginable promise for subpopulations of patients who struggle with relatively rare conditions. All stakeholders must accept responsibility for cutting through the politics to clear the pathway and get the right products and new technologies to the right people at the earliest opportunity. It was with this in mind that we worked with the developers of RELiZORB to convene the national expert advisory board meeting described herein.

## Introduction

### Overview of exocrine pancreatic insufficiency (EPI) and related issues

The pancreas, an essential part of the gastrointestinal (GI) system, is functionally composed of exocrine and endocrine constituents. Because there is minimal anatomic separation of the exocrine and endocrine pancreatic function, disease states that cause disruption or damage in one component of the organ may lead to defects in the other component.^8^

EPI is a relatively uncommon but severe condition that is associated with a variety of complex disorders such as cystic fibrosis (CF), pancreatic cancer, gastric/pancreatic surgery, short bowel syndrome, and chronic pancreatitis. Physiological and biochemical effects of EPI include decreased production and secretion of lipase, increased lipase destruction and degradation, and GI motility disorders. The deficiency in pancreatic enzymes (lipase, amylase, protease) results in impaired digestion and subsequent absorption of all nutrients, fats and fat-soluble vitamins being the most clinically relevant.^8^

Steatorrhea, diarrhea, weight loss, abdominal discomfort, and bloating are hallmarks of EPI. In addition to lifestyle modification (eg, alcohol abstinence, frequent low-volume meals), the typical treatment for EPI is pancreatic enzyme replacement therapy (PERT), the clinical efficacy of which is inconsistent.^9^ Left untreated, or inadequately controlled, the condition leads to increasingly severe GI symptoms, reduced caloric intake, and inability to gain and/or maintain weight, placing the patient at risk for malnutrition.

Malabsorption of fats dramatically increases the risk for malnutrition in patients with EPI.^10^ Because malnutrition further compromises treatment for the medical conditions underlying EPI, enteral nutrition (EN) becomes a vital therapeutic intervention for stabilizing body mass index (BMI) in some cases.^3^ EN formulas contain fats as well as carbohydrates, proteins, vitamins, and minerals to help prevent or address nutritional deficiencies related to malabsorption.

Nutrition support therapy is more complex than commonly appreciated, particularly when prescribed for patients with EPI. Severely pancreatic insufficient patients are unable to digest and absorb long-chain triglycerides contained in EN formulas – particularly healthy fats such as docosahexanoic acid (DHA) and eicosapentaenoic acid (EPA). Currently, there is no published evidence to support the use of any Food and Drug Administration (FDA)-approved PERT products in enteral feedings, and existing clinical guidelines do not support administration of enzymes by mixing them into EN formulas.^3^ Factors such as care settings and nutritional formula types must be considered on a case-by-case basis, and complications must be anticipated and avoided; for example, the use of crushed PERT capsule contents in enteral feedings, or capsule contents administered directly though the feeding tube, causes tube clogging and requires frequent monitoring.^11^

From the patient perspective, some with CF rely on nocturnal EN for adequate caloric intake to maintain and hopefully improve BMI and lung function. Following pancreatic and other GI surgeries, EN is essential for patients who experience gastroparesis and are unable to tolerate oral meals. Lacking an appetite for food, patients with pancreatic cancer often use EN to prevent cachexia. EN is helpful for some patients with cerebral palsy who experience pain while eating solid food.

### Novel approach to addressing challenges of nutritional support therapy for patients with EPI - *Eric First, MD, Alcresta Therapeutics*

For the population of patients who require EN because they lack the natural ability to sufficiently hydrolyze and absorb dietary fats, there is a compelling need that is inadequately addressed by current medical practice. Alcresta Therapeutics developed a unique enzyme-based hybrid product in order to fill this treatment gap. The RELiZORB (immobilized lipase) enzyme cartridge focuses on the critical need for fat hydrolysis in EN when administered to patients with EPI. The product is specifically designed to address the challenges faced by patients living with serious conditions such as CF, pancreatitis, pancreatic cancer, and severe digestive disorders. Indicated for use in pediatric patients (ages 5 years and older) and adults to hydrolyze fats in enteral formula, the product is now used by more than 63 US hospitals primarily for patients with CF who receive EN. CF affects ∼30,000 children and adults in the United States, and ∼11% of these patients require EN to increase energy intake in hopes of increasing weight and BMI, both of which are correlated with pulmonary outcomes (lung function expressed as FEV1).^12^

### Purpose of expert advisory board meeting

By improving care efficiency and clinical outcomes for patients receiving nutrition support for EPI, the digestive enzyme cartridge also assists in improving the quality of care and patient quality of life. Both clinical studies and real-world evidence have demonstrated the product's effectiveness as a chronic condition management tool for this complex population of patients who do not have a viable alternative.

Information regarding the value of this product and the consequential implications under new reimbursement and care delivery paradigms has yet to reach mainstream health care providers and payers. Chief among the challenges are payer issues (eg, “lack of medical necessity” and “investigational” determinations, prior authorization and appeal requirements, adjudication as a device rather than a pharmaceutical). The Centers for Medicare & Medicaid Services bundled the product under an enteral supply billing code when it was launched; however, as of May 2018, only 25% of the cost was reimbursed for patients with private insurance. Alcresta is working to help patients maintain access to the product.

An Expert Advisory Board Meeting was convened jointly by Alcresta, Inc., and the Jefferson College of Population Health to increase awareness of the potential value of the product – from a clinical quality, cost-effectiveness, and patient outcomes perspective – among important stakeholder communities and engage experts in constructive dialogue around broadening the dissemination of information to providers and payers.

### Expert advisors

**Joseph Boullata, PharmD, RPh, FASPEN, FACN** - Clinical Professor in Nutrition Sciences at Drexel University; Pharmacy Specialist in Nutrition Support at Hospital of the University of Pennsylvania; research and publications in the areas of nutrition, gastroenterology, and critical care.

**David C. Evans, MD, FACS, PNS** - Director of Nutrition Support Services, Trauma Medical Director, and Associate Professor of Surgery at The Ohio State University Wexner Medical Center in Columbus, OH.

**Robert S. Gregory, RPh, MS, MBA -** Consultant with extensive industry knowledge and experience in managed care, long-term care, and hospital pharmacy; recent managed care organization experience as Pharmacy Director for Aetna Pharmacy Management.

**Winifred S. Hayes, RN, PhD, ANP** - Founder, President, and CEO of Hayes, Inc.; Board of Directors for Utilization Review Accreditation Commission (URAC); first President of the National Association of Independent Review Organizations.

**Jeffery Lerner, PhD** - President Emeritus of Emergency Care Research Institute (ECRI), a designated Evidence-based Practice Center by the US Agency for Healthcare Research and Quality and listed as a Patient Safety Organization by the US Department of Health & Human Services.

**Edith Mitchell, MD, FACP, FCCP** - Researcher in pancreatic cancer and other GI malignancies whose work involves new drug evaluation and chemotherapy, development of new therapeutic regimens, chemoradiation strategies for combined modality therapy, patient selection criteria, and supportive care for patients with GI cancer.

**Gary M. Owens, MD -** Strategic and tactical consulting services to pharmaceutical manufacturers, new technology developers, employer benefit managers and managed care plans.

**Leslie Schechter, PharmD -** Advanced Practice Pharmacist specializing in Pain Management and Nutritional Support at Thomas Jefferson University Hospital.

**Joseph M. Sinopoli, RPh** - Managed Care Pharmacy Director/Formulary Ops Specialist assisting with the formulary development, reimbursement and contracting process; currently working with a large regional health plan and a major pharmacy benefits manager.

**Virginia A. Stallings, MD** - Professor of Pediatrics at the University of Pennsylvania Perelman School of Medicine; Cortner Chair in Gastroenterology and Nutrition and Director of the Nutrition Center at The Children's Hospital of Philadelphia, Philadelphia, PA.

**Kay Vavrina, RD, LD, CNSC** - 20 years' experience in clinical dietetics with more than 8 years specializing in Cystic Fibrosis Nutrition; University of Texas Health, San Antonio Cystic Fibrosis Center; lecturer in Nutrition and Biology departments at the University of Texas San Antonio.

## Meeting Proceedings

On May 11, 2018, a panel of experts was convened at the Jefferson College of Population Health in Philadelphia, PA, to provide multi-stakeholder perspectives on this innovative product in terms of its potential role in optimizing clinical and cost outcomes for the population of patients who require EN support for the treatment of EPI.

### Understanding nutrition support and nutritional pharmacotherapy – *Joseph Boullata, PharmD, RPh*

Nutrition support therapy is a complex process – from patient assessment and prescribing, to administration and monitoring – involving multiple providers, thereby increasing the risk for errors. In general, the goal of nutrition support including EN is to prevent or address malnutrition in patients who are unable to eat or meet daily energy demands via oral intake alone.

Malnutrition is an acute, subacute, or chronic state of nutrition in which a combination of varying degrees of overnutrition or undernutrition, with or without inflammatory activity, have led to a change in body composition and diminished function. Affecting 20%-50% of hospitalized adult patients, malnutrition is associated with untoward consequences including increased length of stay and resource utilization and heightened risk for readmission, infection, and overall mortality.^13,14^ A state of malnutrition is routinely documented in the presence of 2 or more of the following: unintentional weight loss, decreased muscle mass, decline in functional status, insufficient energy intake, decreased subcutaneous fat mass, and accumulation of fluid.^15,16^

EN refers to the system of providing nutrition directly into the GI tract, bypassing the oral cavity. An important therapeutic intervention for a variety of clinical indications, EN includes adult, pediatric, and infant nutrient formulas. Appropriate use of EN incorporates practices that maximize patient benefit while minimizing adverse events. Although it is commonly associated with acute care, rehabilitation, and long-term care settings, ∼438,000 patients use EN in the home setting; one third of these patients use it for ≥5 years.^17^ This often includes administration during the night to allow freedom of movement during the day.

The EN intervention begins with the selection of a formula to meet patients' energy, protein, and fluid needs relative to their clinical status. The formula recommendation is fully documented and discussed with the prescriber and treatment team. Once implemented, EN must be monitored for clinical complications and frequent challenges. For example, when best practices are not followed, feeding tubes can easily become clogged with EN formula and/or drug residue.^18^

Any alteration of a commercially available drug dosage form affects the timing of drug delivery into the GI tract and its clinical effect. Ideally, enteral medications should be prepared in the pharmacy but, in practice, they often are prepared by hospital nurses or patients/caregivers in the home setting. The potential consequences of drug handling errors (eg, unsuitable drug formulation, improper preparation method) can lead to serious consequences – from tube clogging and therapeutic failure to drug toxicity and death.^18^ Although nearly all of those who prepare and administer drugs via EN are confident that their technique is appropriate and effective, observational studies show that error rates approach 60%.^19^

For example, patients with CF requiring PERT are often prescribed an oral PERT product, sometimes referred to by its generic term pancrelipase. Like similar FDA-approved products, pancrelipase is a delayed release capsule. The product should not be crushed and, if granules are added directly to enteral formula, the liquid binds to the enteric coating and the feeding tube becomes clogged. To decrease the incidence of clumping, some resources recommend suspending the granules in thickened water, juice, or applesauce.

Mixing granules with EN formula may cause the enzymes to begin activating prior to administration.^11^ Dissolving granules in sodium bicarbonate solution is sometimes effective, but there is no evidence that this approach improves the breakdown and absorption of nutrients in EN formulas.^11,20^ The result in each case is the inadequate delivery of nutrition to the patient.

### The clinical science of RELiZORB – *Archie Stone, PhD*

Malnutrition often occurs secondarily to other conditions. Effective treatment of malnutrition helps patients cope with their primary conditions. Aside from intestinal failure, CF is the chief, and perhaps the most challenging, of the conditions with secondary risk for malnutrition because of EPI and fat malabsorption that are associated with the condition.

Published data from the CF Foundation patient registry in addition to numerous publications dating back to the 1980s have suggested a strong relationship between lung function and a healthy BMI in patients with CF.^21^ The data show that maintaining a healthy nutrition status is critical to maintaining or improving lung function for these patients. As nutrition status deteriorates (measured in terms of BMI), a patient's lung function decreases, thereby increasing the frequency and severity of pulmonary exacerbations that result in hospitalization and increased health resource utilization.^22^ Health care staff at today's CF treatment centers are tasked with identifying and addressing signs of malnutrition in patients with CF at every clinical visit. Although oral meals, snacks, and supplementation are adequate to meet the daily caloric demands for a majority of these patients, ∼11% become malnourished and require overnight EN to achieve a healthy BMI.

**FIG. 1.**
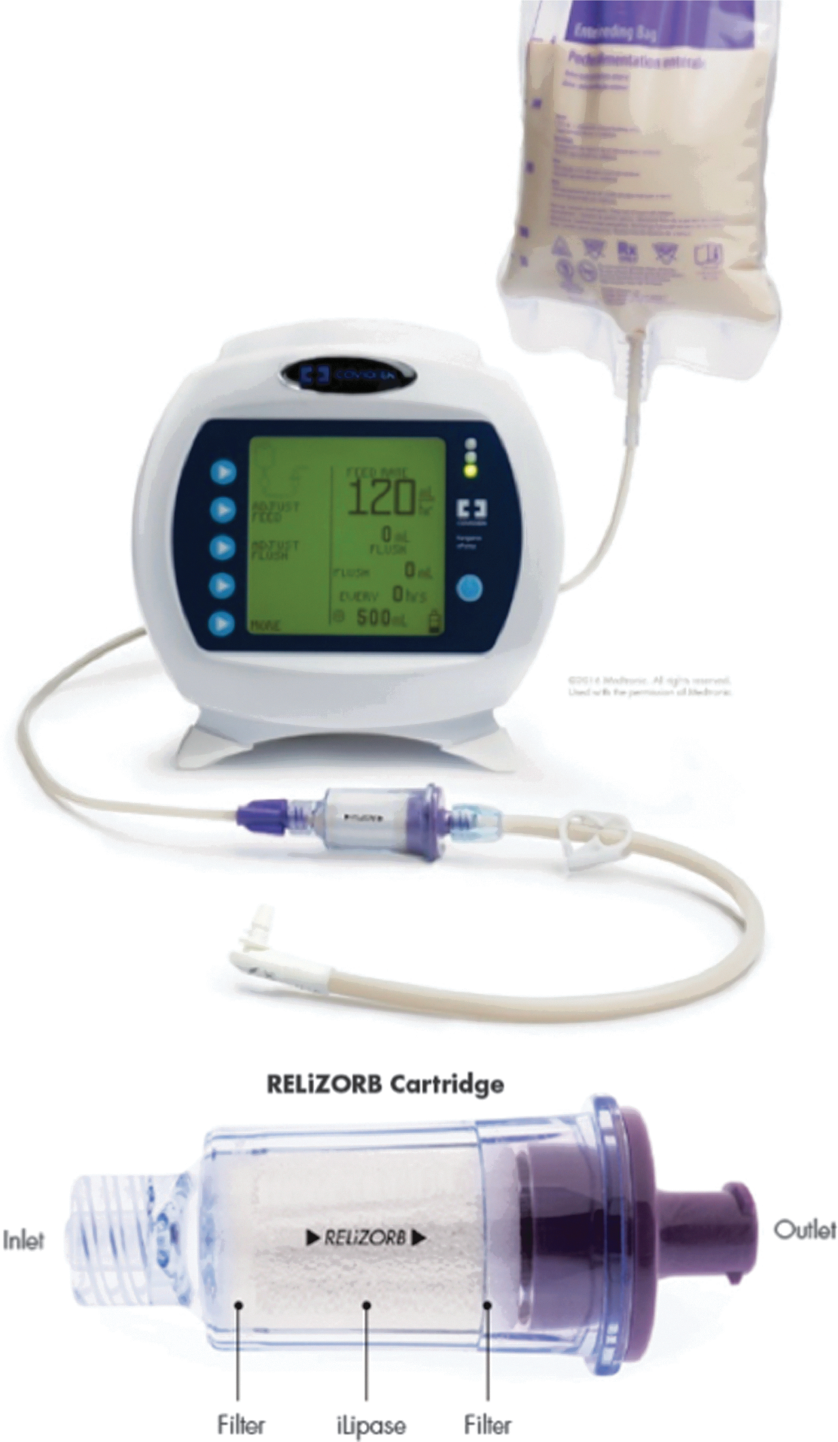
Enteral feeding system and detail of RELiZORB enzyme cartridge.

The RELiZORB immobilized lipase enzyme cartridge was developed to help maximize the effectiveness EN for these patients and other chronically ill patients with EPI-associated conditions. As shown in Figure 1, the cartridge is connected in-line between the formula bag and the feeding tube. This allows the fat hydrolysis to occur ex vivo.

Evidence from 2 prospective clinical trials encompassing ∼2% of the total CF tube feeding population demonstrate normalization of fatty acids in plasma and tissue as well as a reduction in GI events with use of RELiZORB.^4,23^ A longer term 90-day study showed a trend of improved clinical outcomes, with 61% of patients having improvement in weight z-scores and percentiles. In addition, improvement in BMI and reduction of GI adverse events without PERTs during EN feeding has been documented.^23^

**FIG. 2.**
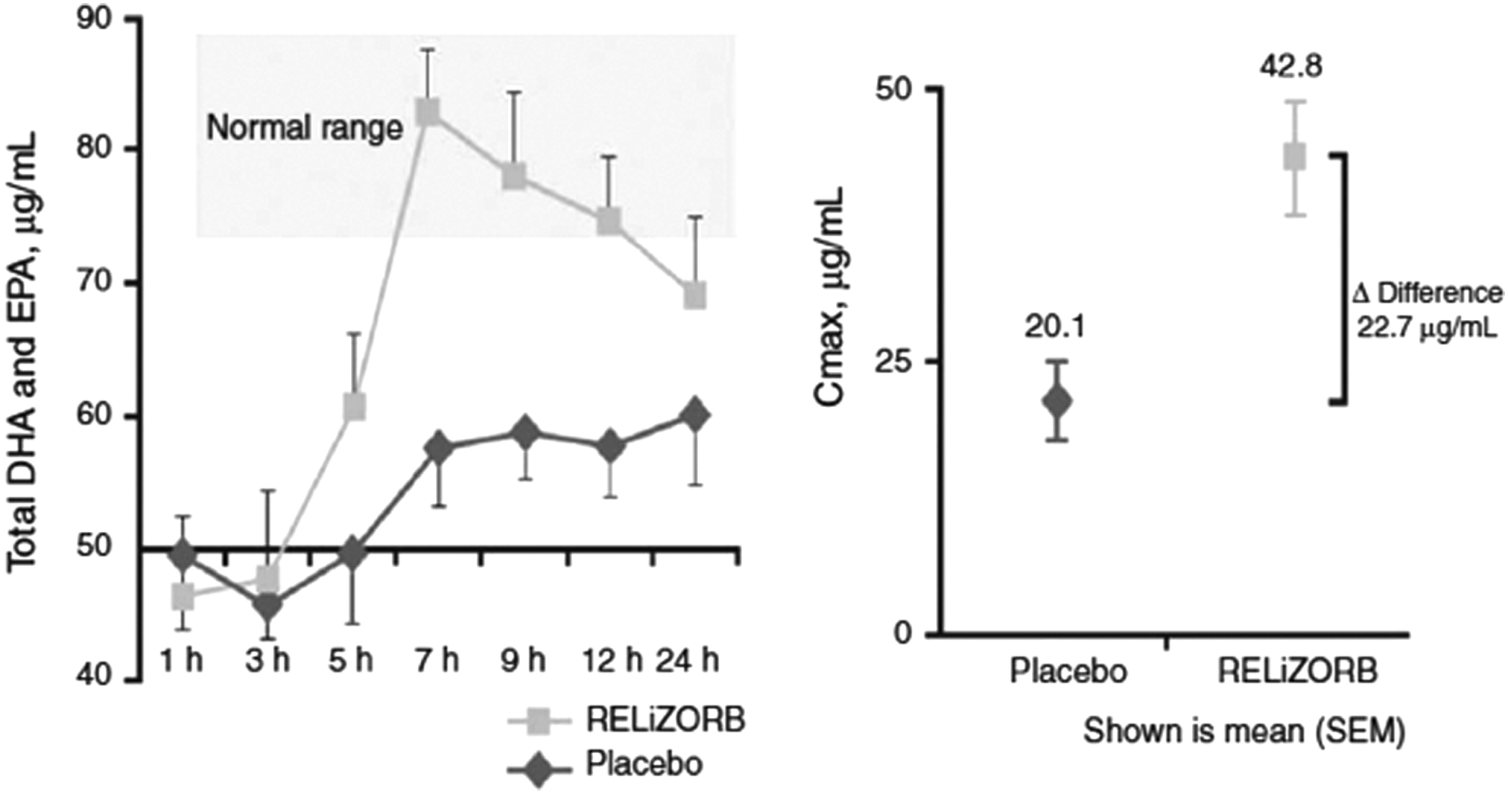
RELiZORB study 497 results: plasma absorption profile and C_max_ for total docosahexaenoic acid (DHA), baseline adjusted. EPA, eicosapentaenoic acid; RBC, red blood cell. Source: Freedman S, Ornstein D, Black P, Brown P, McCoy K, Stevens J, Grujic D, Clayton R. Increased fat absorption from enteral formula through an in-line digestive cartridge in patients with cystic fibrosis. J Pediatr Gastroenterol Nutr 2017;65:97–101. https://journals.lww.com/jpgn/FullText/2017/07000/Increased_Fat_Absorption_From_Enteral_Formula.22.aspx. Reprinted with permission.

*Study 497,*^4^ a multicenter, double-blind, crossover study with an open label safety evaluation period, evaluated the efficacy and safety of the product in CF patients receiving EN. At entry into the study, the 34 patients used on average 8.5 PERT capsules per tube feeding and had baseline fatty acid plasma levels significantly below those of healthy individuals despite receiving EN for a mean 6.6 years. Results showed that use of the cartridge resulted in a statistically significant 2.8-fold increase in fat absorption vs. placebo, fewer reported GI events irrespective of PERT use, and improved tolerability vs. baseline (Figure 2). Investigators reported that use of the cartridge was associated with a 57% reduction in the incidence of diarrhea. In addition, more participants reported preservation of appetite and breakfast consumption when using the digestive cartridge compared with their pre-study regimens. This likely was because of an increase in fat absorption from EN and an associated decrease in the frequency and severity of symptoms of malabsorption.

**FIG. 3.**
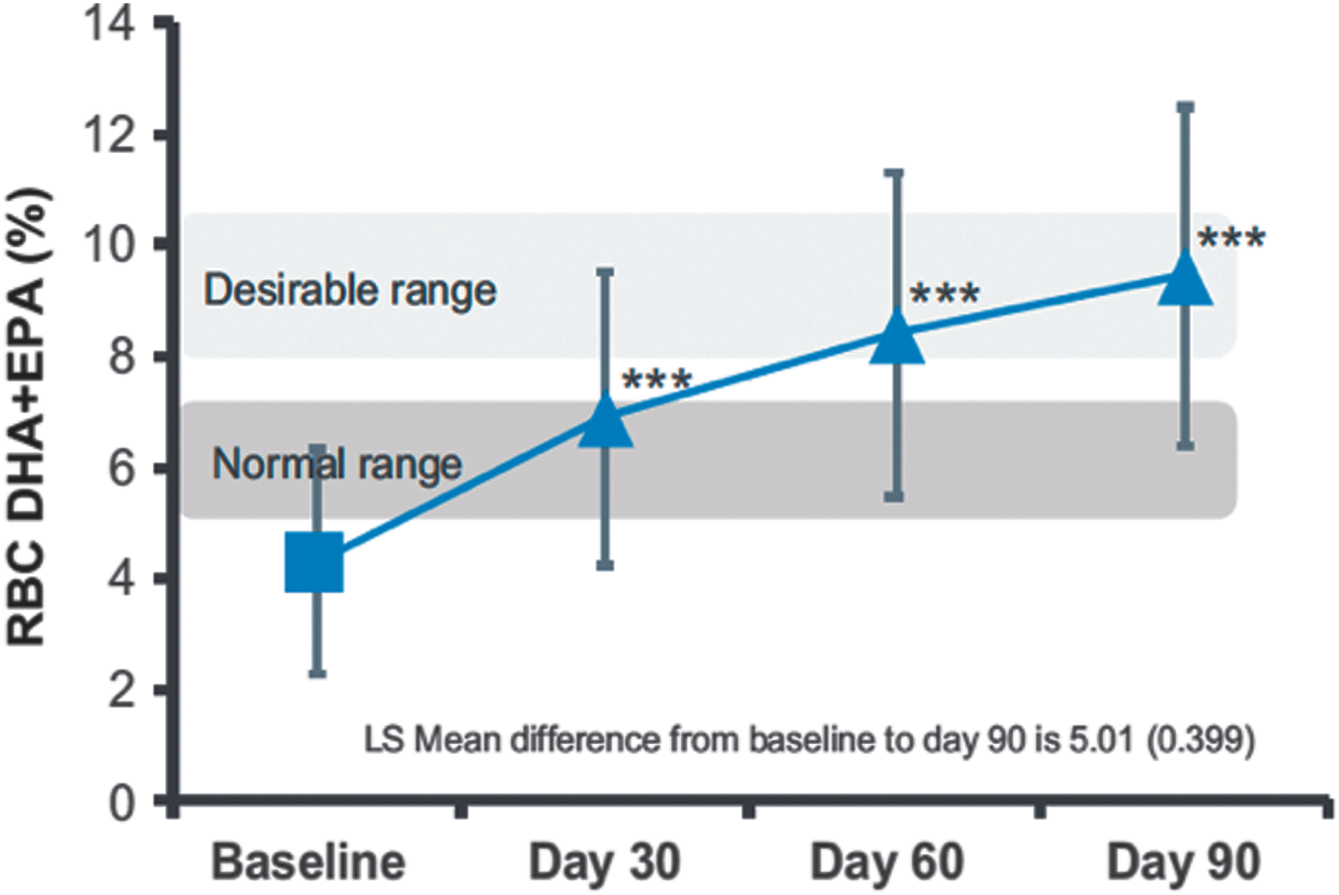
ASSURE Study results: Changes in erythrocyte membrane fatty acid composition (%) for omega-3 index ITT (intention to treat) population as measured by gas chromatography-mass spectrometry. DHA, docosahexamoic acid; EPA, eicosapentaenoic acid; SEM, standard error of mean. Source: Stevens J, Wuatt C, Brown P, Patel D, Grujic D, Freedman SD. Absorption and safety with sustained use of RELiZORB evaluation (ASSURE) study in patients with cystic fibrosis receiving enteral feeding. J Pediatr Gastroenterol Nutr 2018;67:527–532. https://journals.lww.com/jpgn/Fulltext/2018/10000/Absorption_and_Safety_With_Sustained_Use_of.21.aspx. Reprinted with permission.

The *ASSURE Study* (498),^23^ a long-term, 90-day study demonstrated significantly improved fat absorption in terms of increases in red blood cell levels of DHA+EPA (an indicator of tissue uptake), improved omega-6/omega-3 ratios, and increases in plasma levels of DHA+EPA (Figure 3). Use of the cartridge raised omega-3 fatty acid levels in both plasma and erythrocytes (surrogate for tissue uptake) and improved nutritional parameters among children and adults with CF receiving EN. Although the changes were not statistically significant from baseline to 90 days, 61% of patients in the study experienced improvements in weight, and BMI z-scores and percentiles over the course of the study.

*Real-world evidence* is accruing from multiple children's hospitals regarding the effectiveness of the product in improving outcomes for children with CF.

- C. S. Mott Children's Hospital, University of Michigan Health System: A retrospective chart review of 17 patients (age range 1–20 years, average age 11.5 years) prescribed the product for at least 3 months showed improvement in symptoms associated with fat malabsorption in 76% of patients: 53% reported less greasy and odiferous stools; 29% reported fewer abdominal aches; 18% reported less nausea, bloating, and vomiting; and 18% reported less frequent bowel movements. Importantly, a 3-fold improvement in weight-for-age z-score was noted during the period when patients were using the product compared to the 3 months prior to starting therapy. The authors concluded, “The findings show that using an immobilized lipase cartridge is feasible and is associated with clinically significant positive outcomes.”^5^
- Nationwide Children's Hospital: A retrospective chart review of 19 patients (age range 1–29 years, average age 14 years) assessed nutrition outcomes including z-scores for weight and BMI 6 months prior to and 6 months following use of the product. There was a positive but not statistically significant trend in growth rates after starting the product. Caregivers and patients reported that the system was easy to use and had a positive impact on important quality of life parameters (ie, improved appetite the following day, fewer adverse GI symptoms, less sleep interruption [no need to wake to take enzymes]). The authors concluded, “The use of the enzyme cartridge simplifies a mundane process, potentially eliminating the need for additional enzyme replacement during enteral feeds.”^24^
- Medical University of South Carolina (MUSC) Children's Hospital: A retrospective chart review was conducted for 13 pediatric patients (mean age 8.3 years) using the product in place of traditional PERT with overnight EN feedings. The product was started when EN was initiated for 4 of the patients; the other 9 patients had been receiving EN for a mean of 3.4 years before the product was initiated. Although these patients continued to use PERT with oral intake, none of the patients had used PERT in conjunction with overnight tube feedings. Results showed resolution of GI symptoms in 6 of the patients. At 3 months, 10 patients experienced improved BMI or weight-for-length percentiles; at 6 months, 5 patients showed improvement in BMI percentile compared to baseline. The authors concluded that, “RELiZORB use was well tolerated, and promoted weight gain and reduction in GI symptoms of malabsorption in children age 3 months to 16 years, even in the absence of traditional PERT with EN.”^6^

Perhaps the most compelling real-world evidence is documented in the case report of a 2-year-old boy with CF and short bowel syndrome.^7^ Despite all attempts to manage the boy's unusually severe symptoms by traditional means (ie, enteral feedings with crushed PERT products via gastric- or gastro-jejunal feeding tube), he continued to experience symptoms of malabsorption along with poor weight gain and growth. Overnight home nursing assistance was required for his treatment regimen because of poor tolerance of EN feedings.

At 23–24 months of age, PERT was stopped and RELiZORB was initiated. Prior to introducing the cartridge, the boy's weight had never been higher than the 15^th^ percentile. From 23–32 months of age, he gained 3.62 kg, with an improvement in his weight-for-age percentile from the <2^nd^ to the 31^st^ percentile. A concurrent gain in his length improved his length-for-age percentile from the <2^nd^ to the 5^th^ percentile. By 32 months of age, he reached the 82^nd^ percentile for BMI for his age.

### Patient perspective

Via a telephone interview with the expert advisors, a 29-year-old woman shared her experience as a person with CF who requires EN. She reported that being unable to digest food is “painful and very unpleasant.” After resisting it for many years, she opted to try EN when her weight dropped sharply in 2017. At first, she used specialized formulas with PERT every 2 hours. This interrupted her sleep, made her feel too full to eat breakfast, and did not help her gain weight. Now using a standard formula with RELiZORB, she reports that the cartridge is very easy to use, that she sleeps through the night, and that she has more energy for engaging in activities. Importantly, she has achieved her goal weight with a gain of 10 pounds.

Before using the cartridge, she required treatment with corticosteroids and other drugs to improve her lung function. Since using the digestive cartridge, she believes that her “body is now fueled to fight infections” and that her overall health has improved.

### Value proposition of RELiZORB – *Amy Connor, MPH, Jim Gamgort, Alcresta Therapeutics*

RELiZORB is the only FDA-cleared product that hydrolyzes fat in EN formula, thereby maximizing the intended benefit of the feeding (ie, reduced GI events, improved appetite, normalization of critical fatty acid levels, optimal use of other nutrients, improvement in weight gain [demonstrated in clinical trials and real-world evidence], reduction in PERT use). Adoption of the product has been excellent among clinicians working with CF patients in select inpatient settings. Consistently positive patient-reported outcomes are indicative of the product's value to those suffering from EPI.

Although the digestive cartridge appears to be cost neutral versus current treatment practice and the associated reduction in GI adverse events is life-changing for patients, costs continue to escalate and the company must build additional evidence of the product's added value.

Results of an independent analysis clearly demonstrated a cost-savings opportunity compared with current unproven treatment practice (ie, crushed PERT capsules added to EN formula) for inpatient usage. Advisors suggested using hospital/inpatient data to make the case for value. There are hidden costs that may not be readily assessed (eg, additional time for nurses to frequently monitor and clear clogged tubing).

Payment for the product remains challenging. There is concern among payers about the cost – especially for patients on Medicaid or receiving disability benefits – when, in fact, the product may result in long-term savings as a result of improved health status that is secondary to good nutrition. In the absence of formal written policy, payers tend to deny prior authorization requests for the product on the basis of “lack of medical necessity” and being “experimental/investigational.” Experts noted that the appeals process is very expensive for payers. They suggested that the company share their data showing that almost 100% of denials are overturned when appealed in a timely fashion.

### Discussion: key observations and recommendations from the expert panel

The following are key points from expert panel discussions that explored the technology in multiple contexts and considered various approaches to better communicating information regarding this novel product and improving access to it for target patient populations.

#### Nutrition support considerations

Intravenous (parenteral) nutrition is one of the most complicated treatments used by physicians when the enteral route no longer meets a patient's caloric needs. This high-risk therapy is best avoided in favor of EN when the GI tract is available, but even EN must be closely managed to prevent clinical complications and practice challenges. Among the latter, clogged EN feeding tubes are still not rare occurrences; these events should be tracked to assure quality of care.

There is very little provider reimbursement for EN care, and there often is no designated health care provider to oversee its use for patients using EN at home.

#### Product use considerations

Experts agreed that the concept and the delivery mechanism make sense. Real-world evidence has demonstrated that people have gained weight and that their symptoms decreased. The product should be considered for use in all patients with EPI who receive EN for ≥5 years. It will be important to determine how to measure compliance when the product is used on an outpatient basis.

The focus for further investigation should be on identifying all of the conditions associated with EPI (other than CF) in which EN may be indicated (eg, chronic pancreatitis, pancreatic cancer) as well as potential inpatient and intensive care applications. The company should consider collecting and presenting appropriate data at the right professional meetings to communicate evidence of safety and effectiveness. Also consider recruiting an expert to present evidence to select professional audiences and disease-specific patient populations.

#### Understand the entire universe of people who are tube fed

Focusing on the science, consider all conditions associated with, or not associated with, EPI. Because CF is one of the most complex conditions, perhaps the CF data could be extrapolated to other conditions. Pancreatic cancer patients are very sick but their overall GI tracts are relatively healthy. What is the measure for improvement in longevity versus better quality of life? Explore the product's effectiveness for patients with celiac disease, a non-EPI condition, given the underlying factors. Also consider nutritional support in the context of end of life.

Emphasize separate strategies for inpatient and outpatient populations. If use of the product is expanded in the inpatient setting, some aspects will translate to the outpatient setting. Focus on premature infants in terms of weight gain and earlier hospital discharge.

#### Build new patient registries and make use of existing ones to build further evidence

The population of patients with metastatic pancreatic cancer who may benefit from the digestive cartridge can be readily identified via existing pancreatic cancer data registries. Seventy-five percent of these patients are inoperable at the time of diagnosis, and those who undergo chemotherapy prior to surgery may benefit from EN using the product.

Experts agreed that more population-based data would enhance current evidence. A registry function should be a top priority. Options include contacting centers that maintain registries (eg, pancreas tumor registries, post-Whipple surgery registries) and starting a company registry to capture data from all conditions associated with EPI to augment randomized clinical trials and to provide additional insights.

#### Do the best quality research possible

The evidence shows that the product improves nutrient digestion and absorption. From a clinical trial perspective, continue to build the evidence base by designing and conducting longer term studies with outcome measures such as improved weight gain (lean body mass) and improved functionality using simple tools (eg, grip strength, BMI).

There are ample data and real-world evidence showing efficacy of the digestive cartridge when used by patients with CF. Extend research beyond CF and focus on developing peer-reviewed scientific evidence. For example:

- A combined, multicenter retrospective analysis of patient outcomes in conditions other than CF.
- A prospective randomized study incorporating the 2018 standard of care and embedding patient-reported outcomes.
- Patient selection criteria are vital. Use the end point of correcting fat malabsorption across different conditions, and identify patient-focused end points such as weight gain.

Additional comments: Look more closely at the relationship between good nutrition, modulated inflammation, and functional outcome. Also, consider different kinds of studies (eg, workforce savings studies, a survey of why payers care).

#### Work with professional organizations toward developing guidelines and standards of care

Product inclusion in CF treatment guidelines (eg, defining the criteria used to diagnose malnutrition, explaining how to adjust dosage per feeding) is the near-term solution to assuring coverage and providing access for the CF population. The CF Foundation does not update guidelines frequently; propose the development of a supplement to the EN guideline. Also, it would be helpful if the CF Foundation was to write a letter to professional organizations about incorporating the product into guidelines.

As new studies are published, lobby professional organizations and ask payers to have their Health Technology Assessment groups review new data and update their summaries.

Work with gastroenterologists to incorporate relevant information in the GI guidelines. This will lead eventually to the product being included in more hospital formularies.

#### Work with payers

Data showing that denials are regularly overturned on appeal are meaningful to payers. Incorporate these in presentations. Develop prior authorization policy guidelines for the product to share with payers.

In the current fragmented market many payers do not cover the product, and much of the cost is absorbed by the company. Enter into dialogue with select payers about conditional coverage as additional evidence is developed. Work with private payers toward adjudicating the product as a drug rather than a device. (This may not be possible with all payers as some have very clear pathways for claim adjudication based on FDA clearance pathways.)

Help payers understand which of their patient populations would benefit from the product. Clearly define the level of fat malabsorption at which EN is justified (eg, for pancreatic cancer patients). Create sample prior authorization scenarios by which payers can easily identify the subpopulations of patients who would be eligible for the product.

National Committee for Quality Assurance policies require health plans to reassess technologies periodically. Ask payers to update their policies pertaining to EN and add the product to the list of permitted investigational drugs.

Among the approaches that the company might take to make the value case for payers are:

- Increase the number of patients and subpopulations, clarify which patients would use the product, and help payers determine benefit designs.
- Limit payer risk by executing creative guaranteed or “capped,” value-based contracts with meaningful outcome measures. A cost ceiling for patients could be included.
- Consider the value of patient-reported outcomes. Survey nurses regarding the challenges associated with traditional EN and the number of diarrhea episodes that patients with malabsorption of fats experience.

#### Understand and expand the market

Develop good data around growing the market. Introduce the product in the metastatic pancreatic cancer patient population. These patients have difficulty taking large amounts of oral medications with food; they are seeking something to help. Design a short-term outcome study – 75% of these patients already have a feeding tube in place.

Consider broader implications and applications for the product as the research base expands to include chronic pancreatitis, intensive care unit patients, and select oncological conditions.

Reach out to integrated health care delivery systems. The Veterans Administration is interested in working with the private sector and would be a different constituency. Also work toward Medicaid coverage for the product.

#### Engage with natural advocates

Dietitians and nurses are critical team members in the nutrition universe; they are the standard bearers. Dietitians are valuable resources because they see – and refer – all patients.

As nutrition champions, clinicians will begin to order the product once they are aware of it. A quick, easy-to-administer test to screen for malnutrition would be helpful to clinicians.

#### Continue to refine the product

From an engineering perspective, focus on the device design, flow rates, and hydrolysis relative to different populations and different formulations. Importantly, foresee where and how the product will be used 2–3 years from now.

#### Work with patients

As employer groups shift more costs to patients via coinsurance and co-pays, patients are making more decisions. Patients who are given the opportunity to use the product will be fearful of losing access related to payer denials. The CF Foundation offers patients a stipend to help cover co-pays.

Look into the success rate of a pro-patient care “hub” – these are limited with respect to cancer, but incentivize providers to treat patients with CF holistically.

## Conclusion

Although new drugs and technologies are being developed to improve the medical management and clinical outcomes for populations of patients with rare conditions, payment determinations often are based solely on the price tag rather than the value for patients and the potential cost savings associated with improved condition management. The RELiZORB (immobilized lipase) enzyme cartridge was developed for the population of patients who lack the natural ability to sufficiently hydrolyze and absorb dietary fats and who require supplemental EN – specifically, patients with conditions such as CF, pancreatitis, pancreatic cancer, and severe digestive disorders. The enzyme cartridge is the only FDA-cleared product that safely and effectively hydrolyzes fat in EN formula, as demonstrated by clinical trials and real-world evidence.

In May 2018, an Expert Advisory Board was convened. The broad range of stakeholder perspectives included: clinicians specializing in nutrition, pediatrics, gastroenterology, critical care, trauma, long-term care, and oncology; commercial payers such as managed care organizations and self-insured employers; nutrition scientists; and patients. Following a review of the evidence for RELiZORB and the value proposition for the product, the experts recommended taking various approaches to increase appropriate physician prescribing of, patient access to, and payer reimbursement for this unique product that targets a rare but very serious chronic condition.
